# Proteomic Analysis of Polypeptides Captured from Blood during Extracorporeal Albumin Dialysis in Patients with Cholestasis and Resistant Pruritus

**DOI:** 10.1371/journal.pone.0021850

**Published:** 2011-07-14

**Authors:** Marina Gay, Albert Pares, Montserrat Carrascal, Pau Bosch-i-Crespo, Marina Gorga, Antoni Mas, Joaquin Abian

**Affiliations:** 1 CSIC/UAB Proteomics Laboratory, Instituto de Investigaciones Biomédicas de Barcelona-Consejo Superior de Investigaciones Científicas/Institut d'investigacions Biomèdiques August Pi i Sunyer (IIBB-CSIC/IDIBAPS), Bellaterra, Spain; 2 Liver Unit, CIBERehd, Hospital Clínic, Institut d'investigacions Biomèdiques August Pi i Sunyer (IDIBAPS), University of Barcelona, Barcelona, Spain; Biological Research Center of the Hungarian Academy of Sciences, Hungary

## Abstract

Albumin dialysis using the molecular adsorbent recirculating system (MARS) is a new therapeutic approach for liver diseases. To gain insight into the mechanisms involved in albumin dialysis, we analyzed the peptides and proteins absorbed into the MARS strong anion exchange (SAX) cartridges as a result of the treatment of patients with cholestasis and resistant pruritus. Proteins extracted from the SAX MARS cartridges after patient treatment were digested with two enzymes. The resulting peptides were analyzed by multidimensional liquid chromatography coupled to tandem mass spectrometry. We identified over 1,500 peptide sequences corresponding to 144 proteins. In addition to the proteins that are present in control albumin-derived samples, this collection includes 60 proteins that were specific to samples obtained after patient treatment. Five of these proteins (neutrophil defensin 1 [HNP-1], secreted Ly-6/uPAR-related protein 1 [SLURP1], serum amyloid A, fibrinogen alpha chain and pancreatic prohormone) were confirmed to be removed by the dialysis procedure using targeted selected-reaction monitoring MS/MS. Furthermore, capture of HNP-1 and SLURP1 was also validated by Western blot. Interestingly, further analyses of SLURP1 in serum indicated that this protein was 3-fold higher in cholestatic patients than in controls. Proteins captured by MARS share certain structural and biological characteristics, and some of them have important biological functions. Therefore, their removal could be related either to therapeutic or possible adverse effects associated with albumin dialysis.

## Introduction

Extracorporeal albumin dialysis (ECAD) using the molecular adsorbent recirculating system (MARS) has been used in patients with acute-on-chronic liver failure or acute liver failure, and in the treatment of primary graft dysfunction after liver transplantation with favorable, although indefinite, results in terms of survival [1], [2], [3], [4], [5], [6], [7], [8], [9]. Another application of MARS is the treatment of resistant pruritus in chronic cholestatic diseases and in patients with pruritus resulting from liver graft rejection and severe cholestasis. In these latter conditions, the procedure is effective in most patients after only two sessions. Furthermore, albumin dialysis with MARS is well tolerated, and can therefore be considered the last rescue step in patients with pruritus who did not experience favorable results with non-invasive treatments. MARS treatment can avoid liver transplantation in patients with chronic cholestatic diseases who experience severe and unbearable itching [8]. Many substances have been proposed as possible causes of pruritus in cholestasis, including bile salts, steroids and steroid metabolites, histamine and endogenous opioids. However, none of these molecules have been shown to be pruritogens and the pathogenesis of pruritus in cholestasis is still not firmly established [10], [11], [12], [13].

The clinical effects of MARS are presumably due to the removal of albumin-bound substances from the patient's blood [14], [15], [16]. However, the exact mechanism of MARS function and the entire set of substances removed by MARS from a patient's blood remain unclear. Characterization of MARS-captured molecules is hampered by their relatively low concentration in the albumin matrix. In addition, the commercial human serum albumin (HSA) that is used in the MARS circuit contains many other proteins that interfere with the analysis. Recently, we described more than 140 proteins in these preparations in addition to albumin [17]. In MARS, the albumin-enriched dialysate, which contains 20% human serum albumin, is recirculated. Albumin binding sites are regenerated by online perfusion through a strong anion-exchanger (SAX) and charcoal cartridges and the solution is simultaneously dialyzed against a bicarbonate-buffered solution using a standard dialysis machine or hemoperfusion.

**Figure 1:**
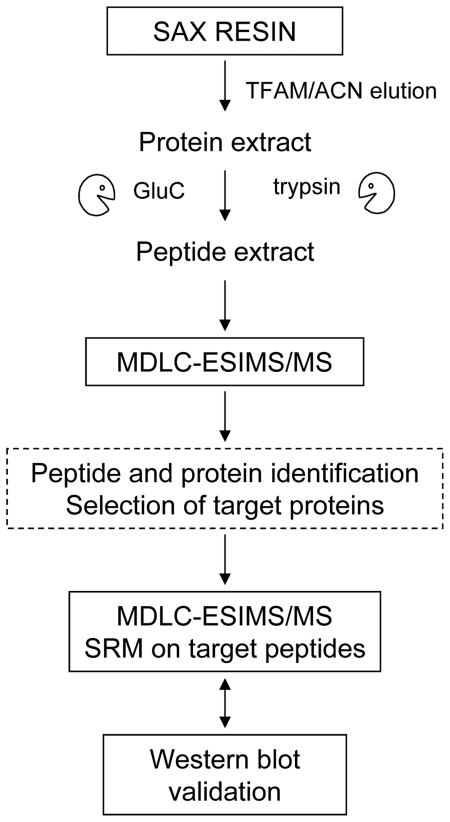
Analytical workflow. Proteins retained in the SAX resin are extracted by stepwise elution. Two aliquots of these extracts are digested with two different enzymes (trypsin and GluC, respectively) and peptides identified by MDLC-ESIMS/MS. Target peptides were confirmed by selected reaction monitoring. The identification of some proteins as removed from patients' blood (and not from the commercial albumin solution used in the MARS circuit) was further validated by Western blot.

In the present study, we used a shotgun proteomic approach combined with targeted MS/MS procedures to describe the set of peptides and proteins that were captured by the MARS system from patients' blood and absorbed in the SAX cartridges in patients treated for resistant pruritus (Figure 1). Our aim was to contribute to further understanding of the role of MARS and its beneficial effects in patients with liver diseases.

## Results

### Peptide and protein identification

Parallel analysis by MDLC-ESIMS/MS of tryptic and GluC (Staphylococcus aureus protease V8) digests of the MARS SAX extracts led to the identification of a total of 1,726 distinct peptides (FDR<1%). Proteins identified from only one peptide were re-evaluated with PEAKS to avoid false positives. Only peptide identifications that coincided in PEAKS and SEQUEST analyses were considered. Using this approach, 1,538 peptides corresponding to 144 proteins were identified with high confidence (Table S1 and Table S2). From the total set of proteins, 126 had at least one peptide that was unique to its sequence. Eighty-four of the proteins were detected in both the albumin control extract and the extract obtained after patient treatment with MARS, while a further sixty proteins were identified only in the patient-derived samples (Table 1).

**Table 1:** Data set of proteins captured by MARS.

Accesion # (Uniprot)	Protein Name	Mr (KDa)	# pep	Sequence Coverage (%)	Accesion # (Uniprot)	Protein Name	Mr (KDa)	# pep	Sequence Coverage (%)
P02671	Fibrinogen alpha chain	95.0	58	32	O15091	Mitochondrial ribonuclease P protein 3	67.3	2	10
P01833	Polymeric immunoglobulin receptor	83.3	17	6	Q9NP84	Tumor necrosis factor receptor superfamily member 12A	13.9	2	13
P06727	Apolipoprotein A-IV	45.4	14	30	O00264	Membrane-associated progesterone receptor component 1	21.7	2	12
P59666	Neutrophil defensin 3	10.2	10	58	Q8N2S1	Latent-transforming growth factor beta-binding protein 4	173.4	2	3
P59665	Neutrophil defensin 1	10.2	9	55	P28223	5-hydroxytryptaminereceptor 2A	52.6	2	7
P14209	CD99 antigen	18.8	7	35	P36955	Pigment epithelium-derived factor	46.3	2	10
P10124	Serglycin	17.6	6	22	P18065	Insulin-like growth factor-binding protein 2	35.1	2	8
P24593	Insulin-like growth factor-binding protein 5	30.6	5	26	P60985	Keratinocyte differentiation-associated protein	11.0	2	11
P10645	Chromogranin-A	50.7	5	14	P00747	Plasminogen	90.6	2	5
P01344	Insulin-like growth factor II	20.1	5	23	P22692	Insulin-like growth factor-binding protein 4	27.9	2	12
Q16610	Extracellular matrix protein 1	60.7	4	12	O00533	Neural cell adhesion molecule L1-like protein	135.0	2	3
P12111	Collagen alpha-3(VI) chain	343.7	4	1	P01298	Pancreatic prohormone	10.4	2	30
P81605	Dermcidin	11.3	4	25	Q15847	Adipose most abundant gene transcript 2 protein	7.8	2	86
P55000	Secreted Ly-6/uPAR-related protein 1	11.2	4	59	Q16661	Guanylate cyclase activator 2B	12.1	2	19
P0C0L5	Complement C4-B	192.8	4	1	P22614	Putative serum amyloid A-3 protein	13.4	1	13
P0C0L4	Complement C4-A	192.8	4	1	P04085	Platelet-derived growth factor subunit A	24.0	1	8
P19022	Cadherin-2	99.8	3	6	Q9ULI3	Protein HEG homolog 1	147.4	1	0
Q96NZ9	Proline-rich acidic protein 1	17.2	3	21	P37837	Transaldolase	37.5	1	7
Q16627	C-C motif chemokine 14	10.7	3	58	Q16819	Meprin A subunit alpha	84.4	1	1
P08493	Matrix Gla protein	12.3	3	30	Q15063	Periostin	93.3	1	1
Q9UHG2	ProSAAS	27.4	3	7	P10451	Osteopontin	35.4	1	4
P02735	Serum amyloid A protein	13.5	3	29	P28335	5-hydroxytryptamine receptor 2C	51.8	1	2
P07602	Proactivator polypeptide	58.1	3	7	P81172	Hepcidin	9.4	1	21
P00746	Complement factor D	27.0	3	14	P54710	Sodium/potassium-transporting ATPase gamma chain	7.3	1	21
P28799	Granulins	63.5	3	10	P35542	Serum amyloid A-4 protein	14.8	1	6
Q16663	C-C motif chemokine 15	12.2	3	31	P08123	Collagen alpha-2(I) chain	129.3	1	1
P30456	HLA class I histocompatibility antigen, A-43 alpha chain	41.0	1	4	Q9Y624	Junctional adhesion molecule A	32.6	1	6
P35527	Keratin, type I cytoskeletal 9	62.1	2	5	P04118	Colipase	11.9	1	12
P02818	Osteocalcin	10.9	2	19	Q8N729	Neuropeptide W	18.0	1	10
P30512	HLA class I histocompatibility antigen, A-29 alpha chain	40.8	1	4	Q8TDB2	Transthyretin amyloidosis variant D38V	4.8	16	60

### Selected reaction monitoring analysis

Twelve of the 60 proteins that were putatively derived from patients were selected for targeted MS/MS analysis in control- and patient-derived extracts. In addition, 5 proteins that were common to both samples were taken as references (Table 2).

**Table 2:** Peptides and proteins monitored by SRM (Full data in Table S3).

Protein	Mr (KDa)	Monitored Peptide	Sample Detected(1)	
			C	P
Alpha-1B-glycoprotein	54.3	SWVPHTFESELSDPVELLVAES	X	X
Serum albumin	69.4	LVNEVTEFAK	X	X
		AVMDDFAAFVEK	X	X
		DYLSVVLNQLCVLHE	X	X
Apolipoprotein A-II	11.2	EPCVESLVSQYFQTVTDYGK	X	X
		KAGTELVNFLSYFVELGTQPATQ	X	X
Neutrophil defensin 1	10.2	EPLQARADEVAAAPEQIAADIPEVVVSLAWDESLAPK	-	X
		ADEVAAAPEQIAADIPEVVVSLAWDESLAPK	-	X
Alpha-2-HS-glycoprotein	39.3	ISRAQLVPLPPSTYVE	X	X
Fibrinogen alpha chain	95.0	TFPGFFSPMLGEFVSETESR	-	X
		FDTASTGKTFPGFFSPMLGEFVSETESR	-	X
Pancreatic prohormone	10.4	AQGAPLEPVYPGDNATPEQMAQYAADLRR	-	X
Serum amyloid A	13.5	SGKDPNHFRPAGLPEKY	-	X
Secreted Ly-6/uPAR-related protein 1	11.2	CKPEDTACMTTLVTVEAEYPFNQSPVVTR	-	X
		SCSSSCVATDPDSIGAAHLIFCCFRDLCNSEL	-	X
Transthyretin	15.9	YTIAALLSPYSYSTTAVVTNPKE	X	X
		ALGISPFHEHAEVVFTANDSGPR	X	X

(1) Detected peptides in control albumin (C) and patient-derived (P) extracts.

Targeted proteins were selected on the basis of their molecular weight and the sequence coverage observed in previous experiments. Thus, we selected proteins of low molecular mass (<15 kDa) that were identified in both replicates with high sequence coverage (>25%). An exception was made for the fibrinogen alpha chain (98 kDa). Although this chain was larger than the MARS membrane cutoff (50 KDa), we selected it as it was identified from a high number of different peptides (58 peptides). This indicates that high amounts of this protein or, more probably, fragments of the protein, are removed during the MARS dialysis process.

**Figure 2:**
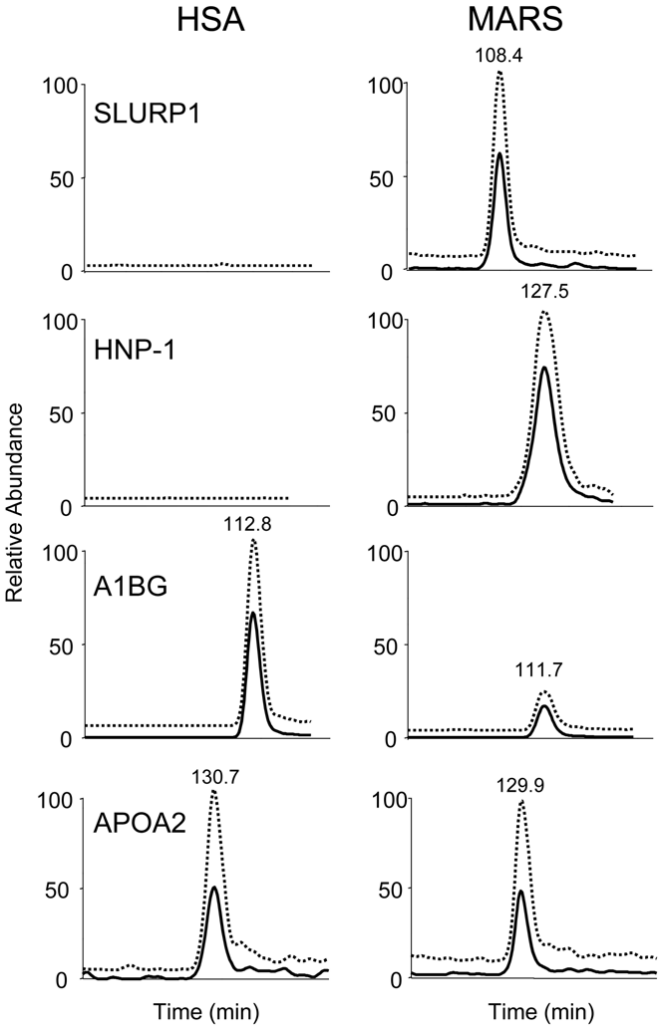
Ion chromatograms for several peptide targets in control human serum albumin (HSA) and patient-derived (MARS) extracts (x-axis range of 8 min). The following SRM transitions were monitored: 1217.5→1298.2, 1441.8 (SLURP1), 1304.4→1739.6, 1080.5, (HNP-1), 1236.6→1183.5, 1515.5 (A1BG) and 1192.9→1969.5, 1683.5 (APOA2) (dotted and solid lines, respectively).

Each targeted protein was monitored through up to four of its previously detected tryptic peptides. These peptides were selected to provide a collection of 34 peptide markers evenly distributed throughout the chromatographic separation. Seventeen of the targeted peptides that represent 10 different proteins were effectively detected and measured by SRM analysis (Table 2). These included five of the proteins that may have been removed by dialysis and five reference proteins (Figure 2). The analyses confirmed that the five candidates in the dialysates came from patients. This set was composed of HNP-1, SLURP1, serum amyloid A, fibrinogen alpha chain and pancreatic prohormone.

### Validation by Western blot

**Figure 3:**
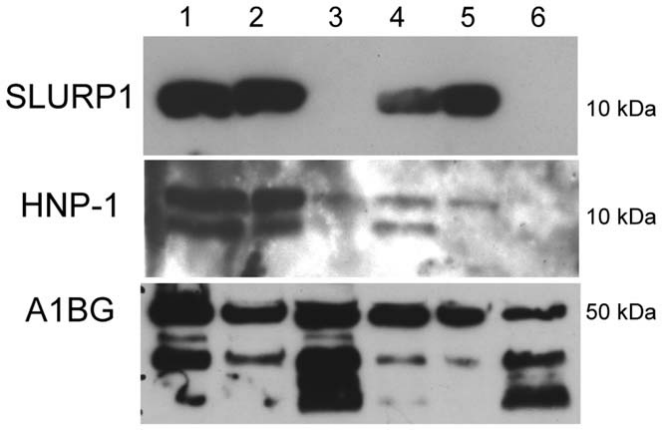
Confirmation of SLURP1 and HNP-1 by Western blot. Lanes 1, 2, 4 and 5 are patients with resistant pruritus; Lane 3 is a patient with Wilson's disease. Lane 6 corresponds to the untreated albumin-derived extracts.

The presence of HNP-1 and SLURP1 was assessed by Western blot on SAX extracts from the five patients treated with MARS. Alpha-1B-glycoprotein (A1BG), which was identified in control and treated samples by MDLC-ESIMS/MS and SRM analysis, was used as a control for the Western blot validation. In agreement with the above-described mass spectrometry results, the presence of HNP-1 was confirmed in all patient samples, but was not detected in the control albumin sample. SLURP1 was not detected in the control sample either. However, it was present in the SAX extract from the four patients with resistant pruritus, but not in the patient with Wilson's disease (Figure 3).

### SLURP1 serum levels in PBC patients and healthy controls

**Figure 4:**
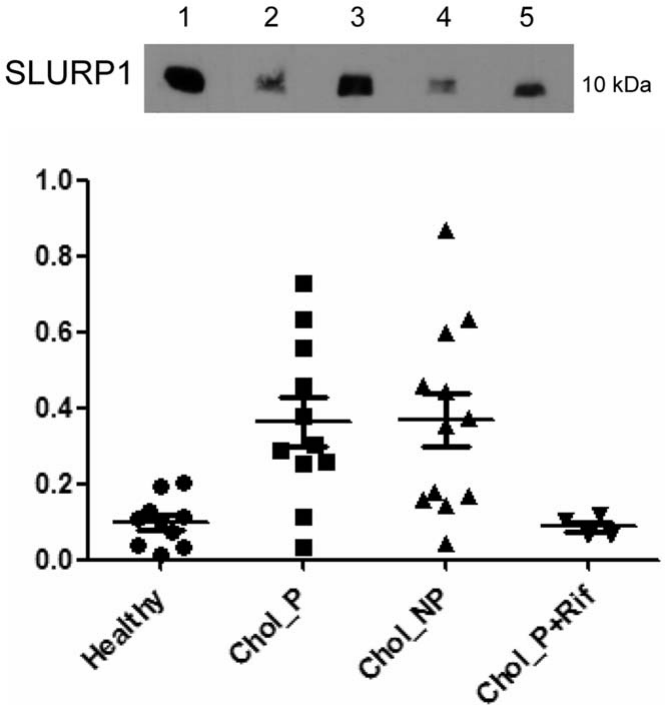
Detection of SLURP1 in serum by Western blot. Lane 1, positive control; Lanes 2 and 4, serum from healthy people; Lanes 3 and 5, patients with cholestasis and pruritus. The graph represents the normalized Western blot intensities of SLURP1 in four groups: healthy people, patients with cholestasis and pruritus (Chol_P), patients with cholestasis without pruritus (Chol_NP), and patients with cholestasis and pruritus who were taking rifampicin (Chol_P+Rif).

One of the SAX cartridge extracts analyzed in the previous experiments was used as a SLURP1 positive control, and the intensities of SLURP1 signals were normalized to this control sample (Figure 4). SLURP1 was identified by Western blot in all control subjects and 27 PBC patients, although the relative concentration was significantly higher in patients (0.32±0.04) than in healthy controls (0.10±0.02) (p<0.005, unpaired t-test, mean ± standard error of the mean). Taking into account the highest level observed in healthy subjects (0.203), 16 out of 27 patients (59%) had high serum SLURP1 levels. SLURP1 was elevated in 9 of the 11 patients with pruritus (82%) and in 7 of the 12 patients without pruritus (58%), but in none of the four patients with pruritus who were treated with rifampicin.

## Discussion

There is still little knowledge of the proteins and other biological compounds that are removed from blood during albumin dialysis. Most of the substances that are known to be removed are small molecules, such as ammonia, bile acids, bilirubin or tryptophan. Several proteins have also been reported to be removed by MARS, including regulators of the inflammatory cascade (tumor necrosis factor-α or interleukin-6) and hepatic growth factors (hepatocyte growth factor or epidermal growth factor). These molecules were identified in studies that analyzed their presence in blood from patients and in the MARS albumin circuit before and after perfusion through the SAX and charcoal cartridges and measured their relative concentrations before and after dialysis [15], [16]. As far as we know, all these measurements were carried out on molecules for which an ELISA, an RIA or an established clinical assay was available. In this study, we performed a global proteomic analysis of the molecules retained in the MARS SAX cartridges, to characterize new peptides and proteins dialyzed from patients' blood. In addition, as many proteins are concentrated in the SAX cartridges during dialysis, our procedure could detect substances in low concentrations that may be difficult to detect at serum levels or in the albumin circuit.

In preliminary experiments, the analysis of proteins absorbed in the MARS cartridges during patient treatment was hampered by the presence of many different proteins that were originally present in the commercial albumin used for the MARS internal circuit. These albumin solutions are industrially prepared from human plasma by the Cohn process [18], which yields albumin of about 95% purity. In a recent study of the composition of this blood derivative, we identified a total of 141 proteins in addition to albumin [17]. The set of proteins detected in these solutions could result from their co-purification with HSA, due to their similar physicochemical properties and/or their affinity for albumin. Interestingly, almost 50% of these proteins have been described previously as forming part of the albuminome [19], [20].

To identify the proteins derived from patients after MARS treatment in these complex albumin extracts, we followed an approach consisting of a shotgun analysis followed by a targeted MS/MS analysis. Molecules identified in the shotgun analysis that had not been identified in control albumin-derived samples were selected for the semi-quantitative targeted MS/MS analysis, in which their presence/absence in control and patient-derived samples was confirmed. For this purpose, we applied an SRM method that provided a high-sensitivity procedure for monitoring the target peptides (Figure 2, Table S3).

Overall, the number of peptides and proteins identified in the analysis of patient-derived samples was similar to that reported in the analysis of control albumin [17]. However, the ratio of non-canonically to canonically-terminated peptides in the patient-derived extracts was twice as high as the corresponding ratio in control albumin (2.1 vs. 1.1, respectively) [17]. This could be due either to efficient, selective transfer of small peptide fragments from patient blood through the dialysis membrane or to protease activity on the material in the dialysis circuit, due to enzymes transferred from blood.

Our search focused on proteins removed from the patients that could not be detected in the albumin control extracts. Other proteins that are originally in the albumin solution may also be removed from blood during the treatment. Identification of these proteins would require the use of isobaric tag for relative and absolute quantitation (iTRAQ) [21] labeling or other similar strategies for semi-quantitative analysis, to detect differences in protein concentrations between samples. Our approach uses a rapid and simple label-free method for characterizing proteins whose concentration considerably changes after dialysis.

**Figure 5:**
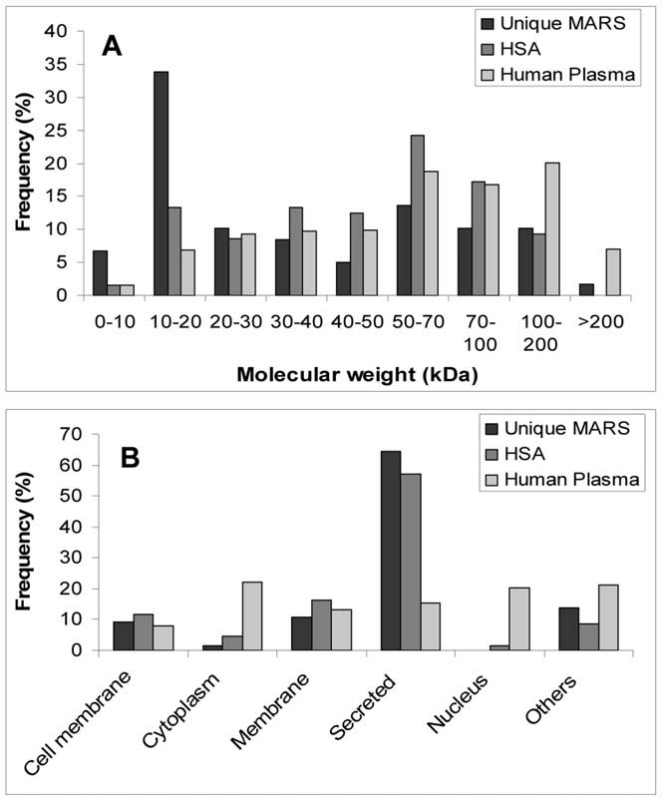
Mass and location distribution of proteins unique to MARS extracts after patient treatment, in comparison with proteins in commercial albumin and in the human plasma database.

The set of proteins that were unique to the extract derived from patient treatment was richer in proteins of low molecular weight than the set of proteins identified in albumin control samples [17] or those expected from the plasma proteome database (http://plasmaproteomedatabase.org) (Figure 5A). A large fraction of these proteins (41%) corresponds to small molecules, with a molecular mass below 20 kDa, that were present in relatively high concentrations (most of the proteins in this group were identified with high sequence coverage and/or a high number of different peptides). This was a direct reflection of the filtering effect of the 50 KDa pore-size dialysis membrane. This group includes HNP-1, SLURP1, matrix Gla protein or C-C motif chemokine-14. Twenty-one proteins (such as plasminogen, granulins, cadherin-2 or extracellular matrix protein 1) with a molecular weight above 50 kDa were also identified. Many of the peptides identified for these proteins did not show tryptic or GluC cleavage at the N- or C-terminus as would be expected from our sample treatment. For example, in the case of fibrinogen, more than 50% of the identified sequences (36 out of 58) did not show tryptic or GluC cleavage. These sequences could correspond to protein fragments dialyzed from blood and in fact, five of the peptides detected had previously been reported as free peptides in human blood (FIBA peptides 24–35, 22–35, 21–35, 20–35, 605–622) [22], [23], [24]. One of these corresponds to fibrinopeptide A, a known cleavage product of the protein. In the case of complement C4 A/B, a high-molecular-weight protein (192 kDa), two of the four sequences detected corresponded to fragments that are not tryptic- or GluC-derived and one of the tryptic fragments had previously been reported as a free protein fragment in blood.

In addition, many of these high-molecular-weight proteins (complement C4-A/B, extracellular matrix protein 1 or polymeric immunoglobulin receptor) were identified with low coverage, although the sequences that justify the assignation were detected with a high number of counts. The number of counts for a peptide (the times it was detected by MS) is related to its abundance in the sample [25]. Furthermore, these sequences could be concentrated in particular regions of the full protein sequence. For example, for complement C4-A/B, for which 95 tryptic peptides were expected from the full protein, we detected only a set of 3 nested peptides covering a zone of 25 aa (1% of the protein) but with a total count of 16 (14+1+1) (Table S1).

Overall, and although it is possible for proteins of non-globular/irregular shapes to pass through membranes with a pore size smaller than its specified MWCO, our data suggest that most of these identifications correspond to sequences derived from protein fragments and not from the full-length protein sequence.

The set of proteins identified only in albumin after dialysis was significantly enriched in proteins with disulfide bonds (DAVID [26], [27] search, fold enrichment = 3.2, p = 2.5E-7) when compared with the proteins in the plasma proteome (http://plasmaproteomedatabase.org). It is interesting to note that a similar enrichment in proteins with disulfide bonds (3.18-fold, p = 2.33E-32) is found in the components of the albuminome described in previous work [19], [17]. Whether these two observations are related and could reflect albumin-affinity-driven transport processes through the MARS membrane is difficult to ascertain from our current data. Additionally, many of these proteins (60%) were found to be interrelated in a STRING (Search Tool for the Retrieval of Interacting Genes/Proteins) [28] analysis, which indicates that they could interact directly or indirectly with albumin (Figure S1). This finding is consistent with the importance of affinity factors in the MARS dialysis process and is in accordance with some hypotheses that explain the MARS effect as a consequence of albumin binding abilities [29].

This set of proteins is significantly enriched in receptor-binding proteins and in proteins that are involved in the inflammatory response (Table S4). Some of the identified proteins show cytokine activity (SLURP1, platelet-derived growth factor subunit A or C-C motif chemokine 15) or are related to the response to wounding (serum amyloid A protein, fibrinogen alpha chain or complement factor D). Secreted proteins were overrepresented (>60%) in the collection of MARS dialyzed proteins in comparison with the distribution expected from the human plasma database. This bias is also observed in the set of proteins found in commercial albumin preparations (Figure 5B).

The capture of the biologically relevant proteins HNP-1 and SLURP1 by MARS was confirmed by Western blot. Interestingly, SLURP1 was not detected in the patient with Wilson's disease, which suggests that its presence could be disease-dependent (Figure 3). Whether SLURP1 is over-expressed in patients with pruritus or under-expressed in the Wilson patient cannot be derived from our data.

SLURP1 is a protein with cytokine activity, which modulates the nicotinic acetylcholine receptor in the presence of acetylcholine [30]. Acetylcholine is related to skin diseases; specifically, it has been described as an inducer of itching in patients with atopic eczema [31]. It has also been suggested that SLURP1 regulates the function of keratinocyte through the cholinergic pathways [32]. SLURP1 has been found to be over-expressed in patients with Mal de Meleda, which is a rare autosomal recessive palmoplantar keratoderma [33].

The quantification of SLURP1 in serum from healthy individuals and patients revealed no statistically significant differences between patients with or without pruritus (Figure 4). However, significant differences were found between patients and healthy people. SLURP1 was overrepresented in patients with cholestasis. A subgroup of the patients with pruritus was treated with rifampicin, a bactericidal antibiotic drug used in the treatment of cholestatic pruritus. This subgroup showed lower levels of SLURP1 than the group of patients with pruritus who were not receiving the drug. The therapeutic action of rifampicin against pruritus has been described as mediated by the nuclear receptor pregnane X (PXR). PXR activates many genes involved in bile acid biosynthesis, detoxification and transportation [34]. Our data suggest that rifampicin could also diminish SLURP1 levels. Further studies with more patients would be needed to confirm such a hypothesis and to establish the mechanisms involved.

In this study, we demonstrated the usefulness of a simple method based on targeted mass spectrometry for characterizing proteins trapped during albumin dialysis in a MARS system. The mechanism of MARS treatments and the set of molecules removed by this system were little known until now. Proteins removed by MARS share some structural and biological characteristics, such as their low molecular weight or their affinity for albumin. Some of these molecules have important biological functions and their removal could be related either to therapeutic effects or to possible adverse effects associated with albumin dialysis. The information reported here constitutes a unique collection of proteins captured by MARS. The study aimed to contribute to the understanding of the effects of albumin dialysis, by assessing potentially disease-relevant molecules removed under the real conditions that occur during treatment. In this respect, we have shown that SLURP1, one of the proteins removed by MARS from patients' blood, is overrepresented in serum from patients with cholestasis.

## Materials and Methods

### Patients and samples

The study protocol conforms to the ethical guidelines of the Declaration of Helsinki and was approved by the ethics committee of Hospital Clínic. All patients gave their informed written consent to participate in the study.

The study was performed with samples taken from blood and MARS SAX cartridges from four patients with cholestasis and resistant pruritus (two patients with primary biliary cirrhosis, one patient with autoimmune hepatitis and one with liver graft rejection) and one patient with a neurologic form of Wilson's disease with no liver involvement who was treated to remove circulating copper. Serum samples from 27 patients with PBC and 10 healthy subjects were also taken and stored at −80°C for subsequent Western blot analysis to validate some of the results of the proteomic analysis of the compounds adsorbed in the MARS SAX cartridge.

### Albumin dialysis treatment

ECAD was performed with a MARS™ system using a constant flow of 20% human serum albumin (Grifols, Barcelona, Spain) in the extracapillary compartment, as described before [8]. A total volume of 600 mL of the albumin solution was used for each treatment and recirculated for 7 h. After the treatment, the SAX column was removed and stored at 4°C until analysis.

### Control sample preparation

Control samples were prepared by recirculating control albumin through a SAX column. For this purpose, a 14-cm long×2.6-cm-i.d. Econo-Pac polypropylene column (20 mL) provided with a frit was filled with 20 mL of resin obtained from a MARS SAX cartridge (diaMARS® IE250, kindly provided by Gambro, Hechingen, Germany). This support is a strong anion exchange (SAX) resin based on cholestyramine. Then, 50 mL of commercially available HUMAN ALBUMIN GRIFOLS® 20% solution w/v were recirculated for 6–7 hours at ca. 30 mL/h. The column was stored for a maximum of 16 h at 4°C before the peptides and proteins bound to the support were extracted.

### Recovery of compounds bound to the SAX support

The overall procedure used for peptide and protein identification is depicted in Figure 1. Briefly, twenty milliliters of SAX resin removed from the MARS cartridges after treatment was placed in 20 mL Econo-Pac columns. In parallel, peptides and proteins absorbed in control and patient-derived supports were extracted. For this extraction, the corresponding columns were washed in 10 mM ammonium acetate, pH 7, and the adsorbed components were eluted by a stepwise gradient of ammonium trifluoroacetate (TFAM, pH 2.5) and ACN (100 mM/5%, 200 mM/5%, 500 mM/5%, 500 mM/20% TFAM/ACN). Six fractions of 30 mL each were collected.

### Sample preparation, protein identification and data analysis

The protein extract derived from one patient with pruritus and the control sample were digested and analyzed as described before [17]. Briefly, two aliquots of the samples were digested with trypsin (acetylated trypsin from bovine pancreas, Sigma-Aldrich) and GluC (*Staphylococcus aureus* protease V8) (Princeton Separations, Inc., Adelphia, USA), respectively. The resulting peptide samples were analyzed by multidimensional liquid chromatography electrospray tandem mass spectrometry (MDLC-ESIMS/MS), using an Agilent 1200 HPLC (Agilent Technologies, Santa Clara, CA) system coupled to a linear LTQ ion trap equipped with a microESI ion source (ThermoFisher, San Jose, CA). Peptides and proteins were identified as described elsewhere [17], [35].

### Selected reaction monitoring analysis

The same MDLC-ESIMS/MS setup as described above was used for SRM analyses. For each of the targeted peptides, the two most intense fragment ions observed in the previous MS/MS analyses were selected for monitoring in the SRM analysis. Ions corresponding to the loss of water from the precursor ion were not taken into account in the selection. The acquisition time for each transition was restricted to a 5–10 min window. Other parameters were: scan time for each SRM event, 3 microscans; maximum injection time, 10 ms; parent ion isolation window, 3 m/z; collision energy, 45%. Collected data on SRM-transitions were evaluated by QualBrowser in Xcalibur 2.0 (ThermoFisher, San Jose, CA).

### Western blot validation

Protein extracts obtained from the SAX MARS cartridges from the four patients with pruritus and the one with Wilson's disease were analyzed by Western blot to confirm the presence of secreted Ly-6/uPAR-related protein 1 (SLURP1) and neutrophil defensin 1 (HNP-1), two of the relevant peptides identified by the proteomic analysis. Moreover, SLURP1 was also assessed in serum samples of patients and healthy controls as follows. To preconcentrate the protein and eliminate interferences in the Western blot analysis, 200 µL of serum were incubated with SAX resin for 4 hours and then extracted with 500 mM TFAM 20%ACN. Equal amounts of SAX-eluted protein were loaded onto a 15% acrylamide∶bisacrylamide gel (MiniProtean, BioRad), and blotted onto polyvinylidene difluoride membranes (Immobilon-P membrane, PVDF, 0.45 µm, 9 cm×6 cm). The proteins were transferred to the membrane at 100 V for 30 min.

The PVDF blots were incubated for 1 hour with blocking solution (5% milk, Nestlé Sveltesse calcium in PBS). Then, three parallel overnight incubations were carried out with primary antibodies for HNP-1 (goat polyclonal HNP C-19, Santa Cruz Biotechnology, Santa Cruz, CA, USA, diluted 1∶100), SLURP1 (mouse monoclonal SLURP1, Abcam, Cambridge, UK, diluted 1∶200) and A1BG (rabbit polyclonal A1BG D-17, Santa Cruz Biotechnology, diluted 1∶400). The three PVDF membranes were washed three times in the blocking solution, incubated with the diluted secondary antibodies for 2 h (anti-goat IgG-HRP, dil 1∶10,000, AbMo rabbit anti-mouse dil. 1∶300, goat anti-rabbit IgG HRP dil 1∶10,000, Santa Cruz Biotechnology) and washed twice in PBS. Staining was then visualized using the SuperSignal West Pico Chemiluminescent Substrate 1∶1 (ThermoFisher, San Jose, CA, USA).

## Supporting Information

Figure S1 Interaction network for 51 of the 60 proteins unique to the MARS extracts after patient treatment that were available in the STRING database. Thirty proteins were found linked at the medium confidence level (score 0.4). Albumin is indicated as ALB (down, center-right).
(TIF)

Table S1 Complete peptide data set identified by Bioworks and PEAKS.
(XLS)

Table S2 Data set of proteins mapped by the identified peptides.
(XLS)

Table S3 Peptides and proteins monitored by SRM. Retention times and SRM transitions.
(XLS)

Table S4 Gene Ontology analysis of the protein set unique to the MARS-derived extracts (DAVID [26], [27]).
(XLS)
