# Supplementary figures and images for: Proteomic Analysis of Polypeptides Captured from Blood during Extracorporeal Albumin Dialysis in Patients with Cholestasis and Resistant Pruritus
Source: PLoS One. 2011 Jul 14;6(7):e21850. doi: 10.1371/journal.pone.0021850 (PMC3136480; doi:10.1371/journal.pone.0021850)

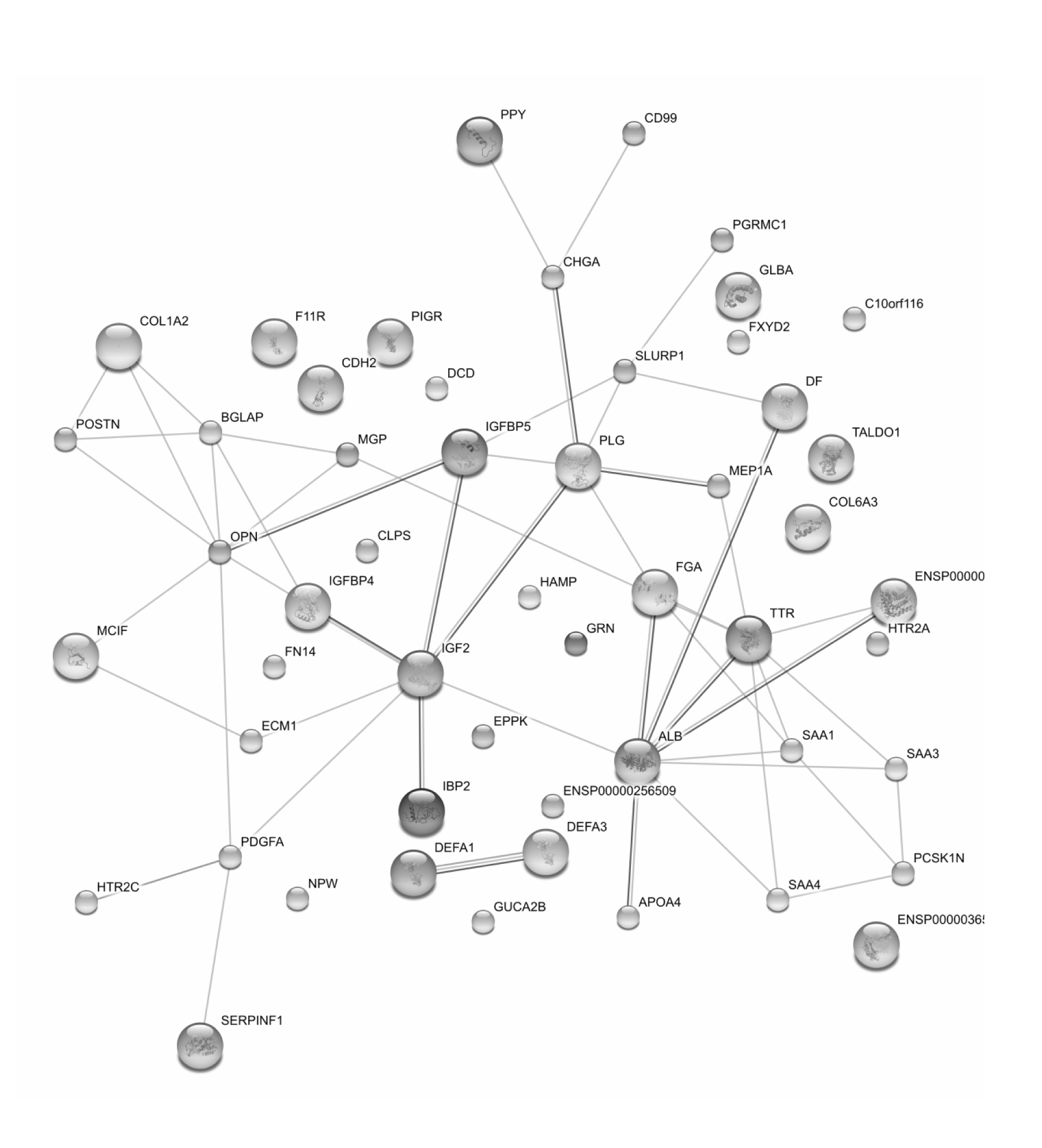

Supplement: Figure S1 — Interaction network for 51 of the 60 proteins unique to the MARS extracts after patient treatment that were available in the STRING database. Thirty proteins were found linked at the medium confidence level (score 0.4). Albumin is indicated as ALB (down, center-right). (TIF) [file pone.0021850.s001.tif]
